# A systematic review and meta-analysis of Comaneci/Cascade temporary neck bridging devices for the treatment of intracranial aneurysms

**DOI:** 10.3389/fnhum.2023.1276681

**Published:** 2023-09-25

**Authors:** Bowen Sun, Shuai Lan, Harshal Sawant, Yuchen Li, Yeping Ling, Bohan Zhang, Pei Wu, Chunlei Wang, Huaizhang Shi, Shancai Xu

**Affiliations:** ^1^Department of Neurosurgery, The First Affiliated Hospital, Harbin Medical University, Harbin, Heilongjiang, China; ^2^Departments of Biomedical Sciences, Joan C. Edwards School of Medicine, Marshall University, Huntington, WV, United States; ^3^Department of Pediatric, The First Affiliated Hospital, Harbin Medical University, Harbin, Heilongjiang, China

**Keywords:** endovascular, intracranial aneurysm, wide-necked, temporary neck bridging device, Comaneci, Cascade

## Abstract

**Background:**

The temporary neck bridging devices represented by Comaneci and Cascade are a type of promising endovascular device for the treatment of intracranial bifurcation or wide-necked aneurysms. This systematic review and meta-analysis aim to assess the efficacy and safety of Comaneci/Cascade devices for the treatment of intracranial aneurysms.

**Methods:**

We performed a systematic literature search on articles in PubMed, Embase, and Web of Science that evaluated the efficacy and safety of Comaneci/Cascade devices for endovascular treatment of intracranial aneurysms, based on the Preferred Reporting Items for Systematic Reviews and Meta Analytics (PRISMA) guideline. We extracted the characteristics and treatment related information of patients included in the study, recorded the rate of technical success, procedural related complications, and angiographic outcomes. The angiographic outcome was evaluated based on Raymond Roy classification, and adequate occlusion was defined as Raymond Ray I + II.

**Results:**

Nine studies comprising 253 patients with 255 aneurysms were included. Among them, eight studies were conducted in Europe, one study was conducted in the USA. All these studies were retrospective. 206 aneurysms (80.78%) were ruptured. The vast majority of patients with ruptured aneurysms did not receive antiplatelet therapy. The rate of technical success was 97.1% (95% CI, 94.9 to 99.3%, I^2^ = 0%). The rate of periprocedural clinical complications was 10.9% (95% CI, 5.4 to 22.1%, I^2^ = 54%). The rate of complete occlusion (RR1) and adequate occlusion (RR1 + RR2) on immediate angiography after the procedure were 77.7% (95% CI, 72.7 to 83.2%, *I^2^* = 35%) and 98% (95% CI, 95.9 to 100%, *I^2^* = 0%) respectively. The rate of complete occlusion (RR1) and adequate occlusion (RR1 + RR2) on the last follow-up angiography were 81.2% (95% CI, 69.2 to 95.2%, *I^2^* = 81%) and 93.7% (95% CI, 85.6 to 100%, *I^2^* = 69%) respectively, with follow-up range from 3 to 18 months. 22/187 (11.76%) cases of aneurysms progressed during the follow-up period. 39/187 (20.86%) cases of aneurysms received additional treatment during the follow-up period. No fatal complications occurred during the treatment.

**Conclusion:**

The Comaneci/Cascade device can be used as an auxiliary treatment for intracranial aneurysms, with a good occlusion effect, but the incidence of complications still needs to be monitored.

## Background

Intracranial aneurysms (IAs) are usually caused by outpouchings of the weak area of the cerebral arterial wall and have always been the main cause of subarachnoid hemorrhage (SAH; Barak et al., 2021; Claassen and Park, 2022). Based on the special hemodynamic state of the bifurcation site of intracranial arteries, aneurysms are prone to form there (Philip et al., 2022). Because it is difficult to treat wide-necked IAs or bifurcation IAs with multiple lateral branches, they have always been considered the Achilles’ heel of endovascular treatment (Webb et al., 2022). Stents and balloons have been widely used for embolization treatment of wide-necked IAs, but they are not perfect. Stents have high requirements for antiplatelet therapy, so they need to be carefully used in the acute phase. Although balloons can be used to treat ruptured IAs, meta-analysis shows that their occlusive effect is mostly inferior to stent therapy at 6 months or later after the procedure (Wang et al., 2016). For bifurcation IAs people have always hoped for the combined application of multiple stents or balloons, which not only improves the occlusion rate of aneurysms, but also increases the incidence rate of ischemic complications (Kuwajima et al., 2022). In recent years, intrasaccular flow disruption devices, mainly including Woven EndoBridge (WEB; MicroVention-Terumo, Aliso Viejo, CA, United States), have been invented specifically for the treatment of bifurcation IAs (Hecker et al., 2023). WEB has been fully applied in multiple studies, with good outcomes and a trend toward expanding indications (Dmytriw et al., 2022; Lee et al., 2023). But currently, it cannot be considered a very mature and perfect device for ruptured IAs.

The temporary neck bridging devices, mainly including the Comaneci device (Rapid Medical, Yokneam, Israel), and the Cascade device (Perflow Medical, St. Netanya, Israel), have attracted a lot of attention in the past decade (Gupta et al., 2016; Sirakov A. et al., 2020; Sirakov S. et al., 2020; Sioutas et al., 2023).^,^ Unlike intrasaccular flow disruption devices, Comaneci and Cascade are metal stents that can be temporarily used for the embolization of IAs, similar in principle to balloon-assisted embolization of IAs (Sirakov et al., 2022). The difference is that the stent structure can ensure the assistance of embolization without affecting blood flow, and compared to the filling state after balloon dilation, the inherent soft structure of Comaneci/Cascade can also reduce the risk of vascular rupture (Taqi et al., 2021). Moreover, Comaneci/Cascade have low requirements for antiplatelet therapy, so they have great potential for safe use in ruptured IAs. A recent study compared the efficacy of Comaneci, stents, and balloons in the treatment of IAs in the acute phase (Taqi et al., 2021). Among them, Comaneci has a lower incidence of hemorrhagic and thromboembolic complications while maintaining a similar occlusive effect to stents and balloons. In addition to ruptured IAs, Comaneci/Cascade is also widely used for unruptured wide-necked or bifurcation IAs, and there is still a lack of comprehensive analysis of their efficacy. Therefore, we performed a systematic review and meta-analysis to assess the safety and effectiveness of the Comaneci/Cascade devices for IAs in the current study.

## Methods

### Search strategy

The current study followed the applicable Preferred Reporting Items for Systematic Reviews and Meta-Analyses (PRISMA) guidelines (Molina-Nuevo et al., 2020). No protocol was registered. We conducted comprehensive literature searches in PubMed, Embase, and Web of Science following librarian recommendations. The time range was from database establishment to 21st April 2023. We used keywords including the following words in combination with Boolean operators to determine the widest search range: “intracranial aneurysm,” “temporary neck bridging device,” “Comaneci,” and “Cascade.” For specific search strategies, please refer to Supplementary Table S1.

### Inclusion and exclusion criteria

We included research articles on the treatment of IAs patients aged ≥ 18 years with Comaneci/Cascade devices. Exclude articles based on the following reasons: (Barak et al., 2021) non-English articles; (Claassen and Park, 2022) Case report; (Philip et al., 2022) Less than 5 cases in the case series; (Philip et al., 2022) Review or editorial articles; (Webb et al., 2022) *In vitro* or animal research; (Wang et al., 2016) Combining the use of other instruments to assist in embolization of aneurysms, such as balloons and stents (Kuwajima et al., 2022). Other unrelated articles. For the articles that ultimately obtained the full text, we evaluated the extractability of the data, population and time of the study, and excluded articles with only abstracts or incomplete information. We retained the latest and most complete cohort for articles with overlapping data.

### Data extraction

Two authors independently extracted the following information from each eligible study: basic information of the study (location, duration, design type), demography information of included patients (number, gender, age), relevant information of treated IAs (location, ruptured or unruptured, various size data, dome neck ratio), treatment information (antiplatelet strategy, technical issues experienced), complications (hemorrhagic and ischemic adverse events and mortality), immediate angiographic outcome, follow-up time, adverse events during the follow-up period, and the follow-up angiographic outcome. A third author resolved the disagreement. According to relevant studies, the angiographic outcome was evaluated based on Raymond Roy classification, and adequate occlusion was defined as Raymond Ray I + II (Fischer et al., 2020; Vinacci et al., 2022). The technical success was defined as successful assistance of the device in the embolization of the aneurysm and covering the neck of the aneurysm, along with perfect removal of the device.

### Qualitative assessment

The study quality was assessed using a modified version of the Newcastle-Ottawa Quality Assessment Scale (NOS; Tomasello et al., 2020). Two independent authors evaluated the quality of each study based on the study group’s selection, the study’s comparability, implementation of results of interest, and follow-up data. A third author resolved the disagreement. Please refer to Supplementary Table S2 for the specific evaluation details.

### Statistical analysis

All statistical analyses were performed using a ‘meta’ package of R software (version 3.6.1, R Core Team, Vienna, Austria). Based on heterogeneity, a meta-analysis was conducted using fixed or random effects to extract proportions from the study, accumulating the occurrence rate and 95% confidence interval (CI) of all outcomes of interest and calculating the cumulative results. Using I^2^ statistics to evaluate research heterogeneity, I^2^ > 50% is considered to have significant heterogeneity and results are calculated using a random effects model. When I^2^ < 50%, use a fixed effect model for result calculation. When a sufficient number of eligible studies formed a funnel plot, visual methods were used to evaluate publication biases. Asymmetric funnel plots indicate publication bias.

## Results

### Systematic research

**Figure 1:**
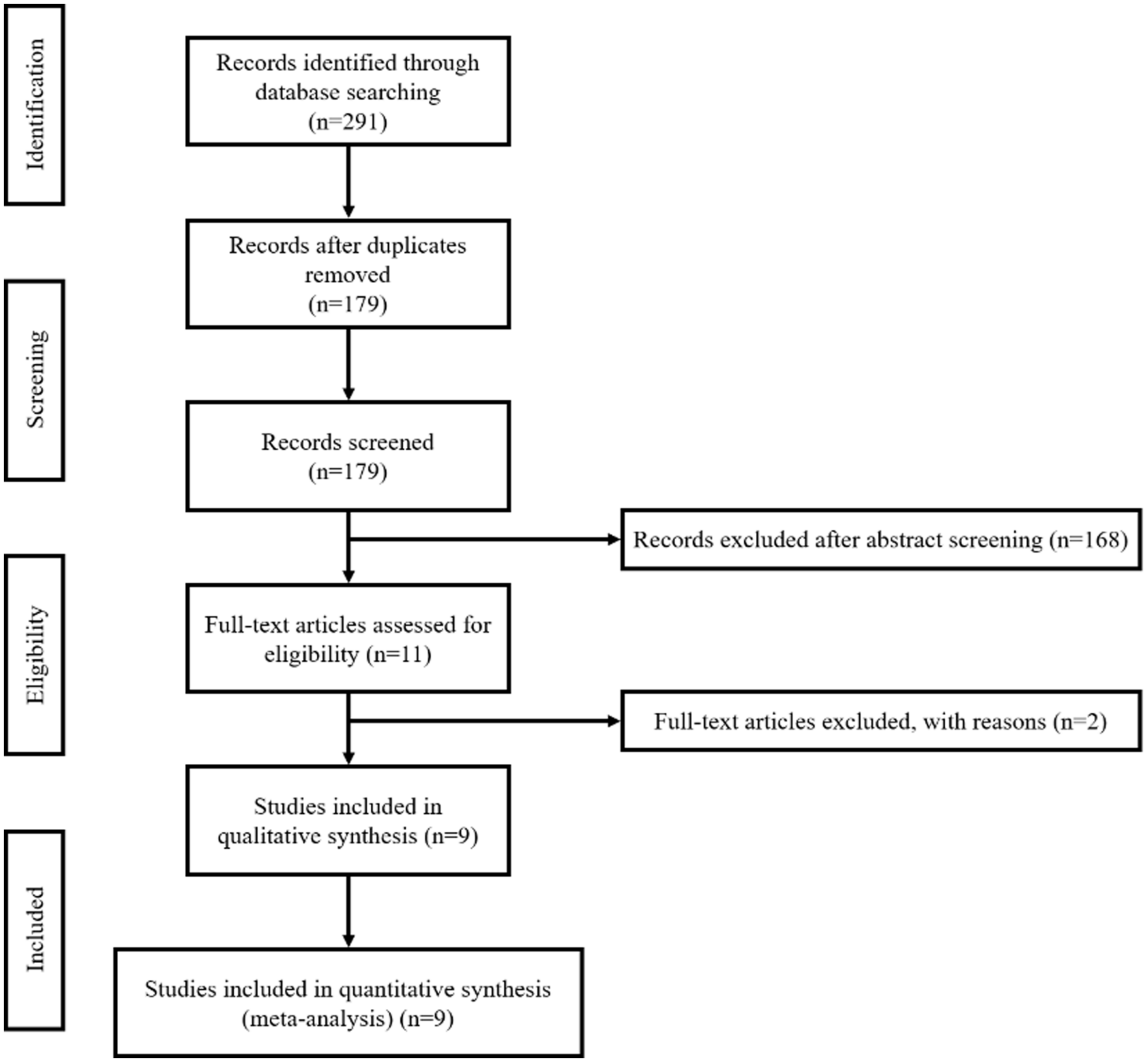
Flowchart of study selection.

According to the strategy, we initially retrieved 291 records, of which 112 were duplicates. We screened the titles and abstracts of the 179 unique records and excluded 168. Among the 11 records that obtained the full text, one was excluded due to data overlap, and one was excluded due to limited information (only an abstract). Therefore, in the final quantitative analysis, we included a total of nine records (Sirakov et al., 2018; Fischer et al., 2020; Molina-Nuevo et al., 2020; Sirakov S. et al., 2020; Sirakov A. et al., 2020; Tomasello et al., 2020; Lim et al., 2021; Taqi et al., 2021; Vinacci et al., 2022). Please refer to Figure 1 for the specific screening process.

### Study characteristics

Out of nine studies, one was conducted in the United States, and eight were conducted in Europe. All the studies were retrospective. Two studies were multicenter. A total of 253 patients were included, the ages ranging from 48.4 to 62.7 years old. Of the 255 IAs included, 206 were ruptured (80.78%). IAs were located more in the anterior circulation, 226 (88.63%) IAs were located in the anterior circulation, and 29 (11.37%) IAs were located in the posterior circulation. The number of internal carotid artery aneurysms was the largest (77/255, 30.20%), followed by anterior communicating artery aneurysms (76/255, 29.80%; Table 1). IAs characteristics were variable among different studies, with diameters ranging from 5.38 ± 2.41 to 6.98 ± 1.71 mm, neck widths ranging from 3.14 ± 1.87 to 4.49 ± 1.93 mm, and dome-to-neck ratios ranging from 1.2 ± 0.3 to 1.77 ± 0.5. Six studies have follow-up records ranging from 3 to 18 months. Each study’s characteristics and outcomes are summarized in Tables 1, 2. Due to all studies being single-arm studies without a control group, their quality is limited. The average score of NOS is 4.6 ± 0.7. The specific evaluated NOS scale can be found in Supplementary Table S2.

**Table 1:** Characteristics of each study included in the current review.

Author, year	Device	Period	Design, country, no. of centers	Sample	Age, mean (yrs. old)	M/F	No. of total aneurysms, ruptured aneurysms	Location	Aneurysm diameter, mean ± SD (mm)	Aneurysm neck width, mean ± SD (mm)	Dome-to-neck, mean ± SD
Fischer et al. (2017)	Comaneci	2014.12–2015.11	R, Germany, 1	18	49.7	13/4	18, 0	ICA, 18	6.0 ± 2.76 (range 2.0–12.0)	3.9 ± 1.6 (range 2.0–8.0)	NA
Sirakov et al. (2018)	Comaneci	2017.5–2017.7	R, Bulgria, 1	29	54.5	16/13	29, 29	AComA, 9; ACA, 1	5.38 ± 2.41 (range 2.7–11)	4.28 ± 1.53 (range 1.9–7.4)	NA
								MCA, 7; ICA, 6; BA, 6			
Sirakov S. et al. (2020)	Cascade	2019.5–2019.6	R, Bulgaria, 1	12	55	7/5	12, 12	Acoma, 3; ICA, 2; MCA, 3; PComA, 2; SCA, 2	6.18 ± 2.08 (range 3–9.1)	4.49 ± 1.93 (range 2.3–7.9)	1.46 ± 0.5 (range 1.06–2.8)
Molina-Nuevo et al. (2020)	Comaneci	2017.3–2019.3	R, Spain, 1	16	48.4	7/9	18, 14	AComA, 2; PComA, 8; ICA, 8	6.02 ± 2.74 (range 2.5–13)	NA	1.73 ± 0.8 (range 0.83–4.30)
Tomasello et al. (2020)	Cascade	2018.7–2019.5	R, Spain, 4	15	58	4/11	15, 5	ICA, 13; VA-V4, 1; SCA, 1	6.27 ± 2.33 (range 2.8–11)	3.64 ± 1.19 (range 1.9–6)	1.77 ± 0.5 (range 0.8–2.4)
Sirakov A. et al. (2020)	Comaneci	2017.8–2019.1	R, Bulgaria, 1	118	55.4	45/73	118, 118	AComA, 45; ACA, 15; MCA, 21; ICA, 24; BA, 5; PCA, 1; SCA, 4; PICA, 3	6.2 ± 4.1 (range 2.6–17.3)	4.31 ± 3.1 (range 2.1–8.6)	NA
Lim et al. (2021)	Comaneci	2019.5–2020.4	R, America, 1	5	58.6	1/4	5, 2	MCA, 3; PICA, 1; OA, 1	6.98 ± 1.71 (range 5.1–9.0)	4.4 ± 0.8 (range 3.6–5.4)	1.2 ± 0.3 (range 0.97–1.6)
Taqi et al. (2021)	Comaneci	2019.7–2020.5	R, America, 4	26	62.7	8/18	26, 15	AComA, 10; MCA, 5; PComA, 4; BA, 3; ICA,1; VA, 1; AchA, 1; SHA, 1	6.59 ± 2.14 (range 3–11.8)	3.91 ± 1.34 (range 1.9–6.5)	1.57 ± 0.45 (range 0.59–3.39)
Vinacci Et Al. (2022)	Comaneci	2015.10–2021.5	R, Italy, 1	14	62.3	5/9	14, 11	AComA, 7; ICA, 5; MCA, 1; PICA, 1	6.77 ± 3.54 (range 2.2–15.6)	3.14 ± 1.87 (range 2–8.2)	NA

No., number; R, retrospective; M, male; F, female; ICA, internal carotid artery; ACA, anterior cerebral artery; AchA, anterior choroidal artery; AcomA, anterior communicating artery; BA, basilar artery; MCA, middle cerebral artery; OA, Ophthalmic artery; PCA, posterior cerebral artery; PcomA, posterior communicating artery; PICA, Posterior inferior cerebellar artery; SCA, supracerebellar artery; SHA, Superior hypophyseal artery; VA, vertebral artery; SD, standard deviation; mm, millimeters; NA, not available.

**Table 2:** Periprocedural and follow-up outcomes of each study.

Author, year	Technical success	Complications	Immediate Occlusion (RR1/RR2)	Immediate RR1	Imaging follow-up percentage	Last image follow-up time (months)	Last image follow-up occlusion (RR1/RR2)	Last image follow-up RR1
Fischer et al. (2017)	14/18, 77.8%	1/14, 7.14%	14/14, 100%	9/14, 64.3%%	11/14, 78.6%	4.8	11/11, 100%	9/11, 81.8%
Sirakov et al. (2018)	28/29, 96.6%	1/29, 3.45%	29/29, 100%	25/29, 86%	29/29, 100%	3	29/29, 100%	28/29, 96.6%
Sirakov S. et al. (2020)	12/12, 100%	0/12, 0%	12/12, 100%	9/12, 75%	NA	NA	NA	NA
Molina-Nuevo et al. (2020)	17/18, 94.4%	4/16, 25%	17/18, 94.4%	16/18, 88.9%	13/18, 72.2%	9.3	NA^*^	NA
Tomasello et al. (2020)	15/15, 100%	0/15, 0%	15/15, 100%	11/15, 73.3%	6/15, 40%	6	6/6, 100%	5/6, 83.3%
Sirakov A. et al. (2020)	115/118, 96.6%	11/118, 9.32%	115/118, 97.5%	83/118, 70.3%	112/118, 94.9%	5.5	93/112, 83%	75/112, 66.9%
Lim et al. (2021)	5/5, 100%	0/5, 0%	5/5, 100%	4/5, 80%	2/5, 40%	3.3 ± 2.5	2/2, 100%	2/2, 100%
Taqi et al. (2021)	25/26, 100%	0/26, 0%	25/26, 96.2%	21/26, 80.8%	NA	NA	NA	NA
Vinacci et al. (2022)	12/14, 86%	5/14, 35.7%	11/14, 79%	6/14, 43%	14/14, 100%	12–18	12/14, 85%	10/14, 71%

RR1, Raymond Roy classification 1; RR2, Raymond Roy classification 2; NA, not available. *Unable to obtain a specific proportion of occluded aneurysms from the original article.

### Technical experience

**Figure 2:**
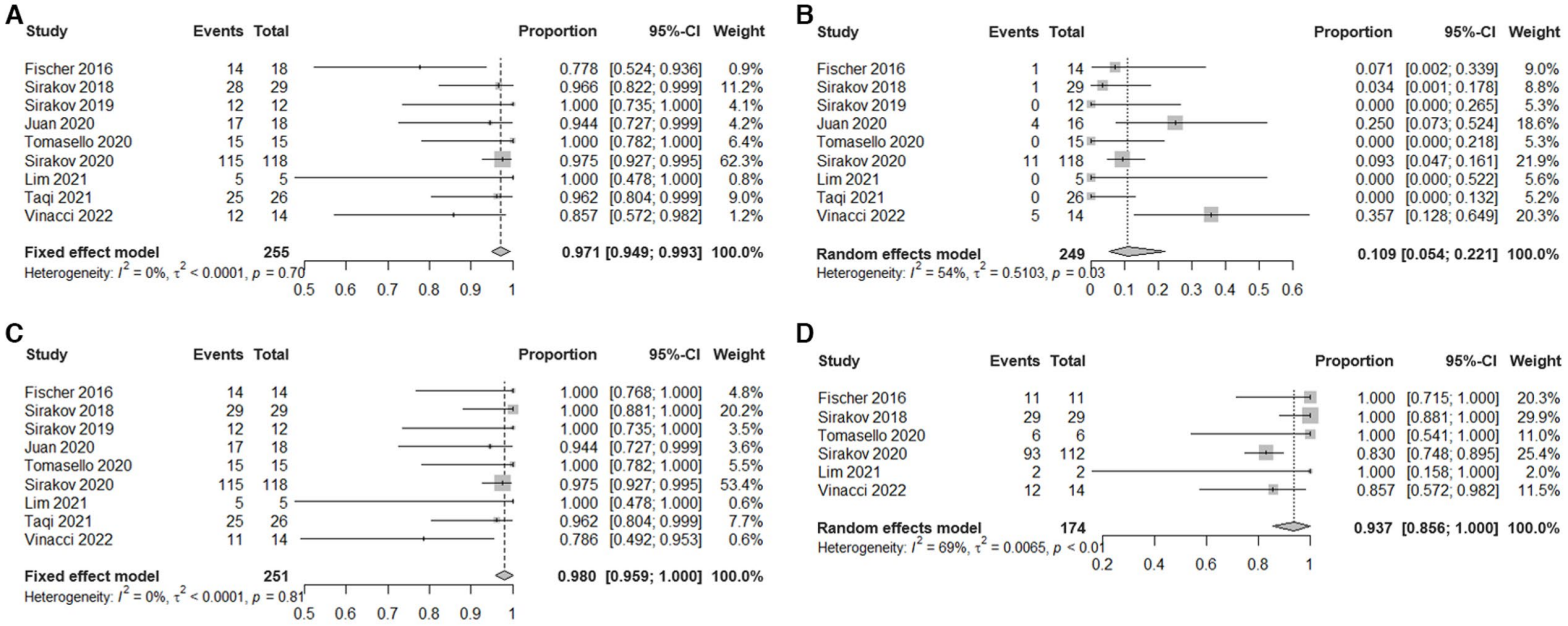
Estimated plotted rates of: **(A)** technical success, **(B)** periprocedural complications, **(C)** immediate adequate occlusion (defined as Raymond-Roy class 1 + 2), and **(D)** Last follow-up adequate occlusion.

Meta-analysis showed that the rate of technical success was 97.1% (95% CI, 94.9 to 99.3%, I^2^ = 0%; Figure 2A). Eight patients experienced technical malfunctions due to unstable devices or inability to achieve dilation and coverage of vascular walls and aneurysm necks. As a result, they required replacement with alternative treatment devices. Severe vasospasm prevented the successful completion of the procedure in one patient. Three patients experienced technical failure due to the operator’s mistake in selecting devices of the wrong size. Please refer to Supplementary Table S3 for the details of technical failure. The antiplatelet strategies of each study were not entirely the same. The vast majority of patients with ruptured aneurysms did not receive antiplatelet therapy, and the specific contents have been summarized in Table 3.

**Table 3:** Antiplatelet regimen in each study.

Author, year	Antiplatelet
Fischer et al. (2017)	DAPT with clopidogrel or ticragrelor and ASA at least 1 day before the procedure, and SAPT with ASA 100 mg/d after the procedure for 4 weeks.
Sirakov et al. (2018)	No DAPT was assigned to any of the treated patients. ASA was administrated to one patient for 2 weeks.
Sirakov S. et al. (2020)	None of the patients received any oral or intravenous antiplatelet therapy.
Molina-Nuevo et al. (2020)	Patients with incidental aneurysms received clopidogrel 75 mg/d and ASA 100 mg/d from 5 days before the procedure. In the rest of the cases, no periprocedural antithrombotic drug was indicated.
Tomasello et al. (2020)	Patients with an unruptured aneurysm (*n* = 10)
	6 received ASA 500 mg preprocedural; 1 received a bolus of inyesprin 900 mg during treatment; 6 received DAPT with ASA 100 mg/daily and clopidogrel 75 mg/d for 10 (*n* = 5) or 12 (*n* = 1) days postprocedure; 1 received ASA150 mg/d for 1 month; and 1 received clopidogrel 150 mg/d for 10 days.
	Patients with a ruptured aneurysm (*n* = 10)
	3 received a bolus of inyesprin (none of the 3 received post-procedural DAPT therapy), and 2 received only heparinization.
Sirakov A. et al. (2020)	No SAPT/DAPT was assigned to any of the treated patients.
Lim et al. (2021)	After the procedure, patients who underwent elective unruptured coiling were maintained on a DAPT (ASA 81 mg/d and clopidogrel 75 mg/d) for 3 months. If follow-up imaging and angiography did not show residual aneurysm, patients were transitioned to a SAPT (ASA 325 mg/d).
Taqi et al. (2021)	Patients with unruptured aneurysms: Aspirin and either clopidogrel or apixaban were provided. Eight patients were treated with DAPT. Two received only ASA monotherapy, and 1 patient did not receive any antiplatelet therapy. Any antiplatelet therapy was discontinued after a successful procedure.
	Of patients with ruptured aneurysms, 10 received no antiplatelet therapy and 5 received ASA only.
Vinacci et al. (2022)	Patients with unruptured aneurysms: DAPT with ASA 100 mg/d and clopidogrel 75 mg/d 5 days before the procedure. The DAPT was discontinued after the procedure if the Comaneci-assisted embolization was successful.
	In patients with ruptured aneurysms, no preventive antiplatelet therapy was provided.

DAPT, dual antiplatelet therapy; SAPT, single antiplatelet therapy; ASA, acetylsalicylic acid.

### Adverse events

Meta-analysis showed that the overall incidence of periprocedural complications is 10.9% (95% CI, 5.4% to 22.1%, I^2^ = 54%; Figure 2B). The main complication is a thrombotic event that occurs inside the device or in the parent artery. A total of 22 cases occurred, seven cases were asymptomatic, 10 cases were accompanied by ischemic symptoms, and most of them had a good prognosis after receiving antiplatelet therapy. Three cases had permanent complications. In addition, in five cases treatment-related vasospasm occurred, and no treatment-related bleeding events or deaths were observed. Please refer to Supplementary Table S4 for the details of adverse events.

### Angiographic outcomes

Meta-analysis showed that the rate of adequate occlusion (RR1 + RR2) on immediate angiography was 98% (95% CI, 95.9% to 100%, I^2^ = 0%; Figure 2C). And the rate of RR1 occlusion on immediate angiography was 77.7% (95% CI, 72.7% to 83.2%, I^2^ = 35%; Supplementary Figure S1). The rate of adequate occlusion (RR1 + RR2) on the last follow-up angiography was 93.7% (95% CI, 85.6% to 100%, I^2^ = 69%; Figure 2D), and the rate of RR1 occlusion on the last follow-up angiography was 81.2% (95% CI, 69.2% to 95.2%, I^2^ = 81%; Supplementary Figure S2). 22/187 (11.76%) cases of aneurysms progressed and 39/187 (20.86%) cases of aneurysms received additional treatment during the follow-up period. Due to the different evaluation criteria for aneurysms progressed and retreatment, combined analysis could not be performed, please refer to Supplementary Table S5 for specific information of recurrent and retreating IAs.

### Publication bias

No significant publication bias was found in each funnel plot of the results. The details are provided in Supplementary Figures S3–S8.

## Discussion

In the current meta-analysis, we pooled the effectiveness of the Comaneci/Cascade device in the treatment of intracranial aneurysms and, reported occlusion and complication rates. The current research holds significance by offering important insights to prospective operators regarding the safety and efficacy of Comaneci/Cascade devices in the treatment of IAs, which can be compared with existing options for the treatment of IAs.

Due to the influence of hemodynamics, wide-necked and bifurcation IAs incidence rate accounts for more than half of the total IAs, and is easy to rupture, accounting for about 60% of the ruptured aneurysms (Barak et al., 2021; Claassen and Park, 2022; Philip et al., 2022). Traditional endovascular treatment for wide-necked and bifurcation IAs mainly relies on coil embolization, but the effect of simple embolization treatment for them is not ideal, often requiring assistance such as balloons and stents (Webb et al., 2022). Moreover, when only a single device is used to assist in the embolization of bifurcation IAs, the coil state is not stable, and is prone to move to the parent artery, leading to treatment failure (Kuwajima et al., 2022; Webb et al., 2022). Therefore, multiple stents or balloons are commonly used in combination to treat bifurcation IAs (Kuwajima et al., 2022) Although such a strategy can effectively treat bifurcation IAs due to the high occupancy of multiple devices in the host artery, it is easy to cause ischemic complications. Therefore, administrating antiplatelet therapy during the periprocedural period becomes necessary, which sometimes makes it challenging to treat ruptured IAs effectively. Moreover, during treatment, the second stent needs to pass through the gap of the first stent or be placed in parallel with the first stent, which can easily cause the first stent to be misaligned and difficult to implant. There is also a possibility of increased operational errors leading to bleeding (Gupta et al., 2016; Sirakov A. et al., 2020; Hecker et al., 2023).

In recent years, there has been rapid advancement in endovascular treatments for IAs with introduction and application of numerous new equipment and innovative concepts. Especially the specially designed intrasaccular blood flow blocking device represented by WEB and the new auxiliary embolization device represented by Contour (Cerus Endovascular, Fremont, CA) for the treatment of bifurcation aneurysms (Gupta et al., 2016; Sirakov S. et al., 2020; Pranata et al., 2021; Dmytriw et al., 2022; Hecker et al., 2023). Unlike them, Comaneci and Cascade, as temporary neck bridging devices, are relatively unique and can be detached. Due to this characteristic, researchers believe that they could be the best choice to avoid antiplatelet therapy during the acute phase (Gupta et al., 2016). In the current analysis of existing evidence on Comaneci/Cascade devices, when compared to other more established and frequently used devices for the treatment of wide-necked and bifurcation IAs, it becomes evident that Comaneci/Cascade devices have a similar incidence of periprocedural complications (10.9%) and a better immediate occlusion rate (98.0%). Cagnazzo et al. conducted a meta-analysis of 27 studies using dual stents to treat 750 bifurcation IAs (66 ruptures) in a total of 744 patients. The immediate postoperative RR1 + RR2 complete occlusion rate was 82.2% (95% CI, 71.4 to 93%, I^2^ = 92%), the incidence of complications was 8.9% (95% CI, 5.8 to 12.1%, I^2^ = 44%), and the mortality rate was 1.1% (95% CI, 0.3 to 1.9%, I^2^ = 0%; Cagnazzo et al., 2019). Recently, Lim et al. compared the effectiveness of Comaneci-assisted embolization for ruptured IAs with balloon and stent-assisted embolization (Taqi et al., 2021). They found that Comaneci was associated with a lower incidence of bleeding and thromboembolic complications, and exhibited complete occlusion and residual retreatment rates similar to those of balloons and stents (Taqi et al., 2021). A balloon is a widely used temporary device to assist in the embolization of aneurysms, similar in principle to the Comaneci/Cascade devices. At present, it is widely believed that the advantage of Comaneci/Cascade devices compared to balloons is that they do not obstruct maternal artery blood flow, allowing uninterrupted blood flow which reduces the chances of thromboembolic events during the embolization process. In addition, Comaneci/Cascade devices can also avoid the potential risk of vascular rupture during balloon-assisted coiling procedures, which is one of its main catastrophic risks (Sirakov et al., 2018; Taqi et al., 2021).

WEB is currently the most commonly used intrasaccular flow disruption device in clinical practice, designed specifically for the treatment of bifurcation IAs. However, it has low occlusion rate due to its disruption principle. Adeeb et al. treated 683 aneurysms (144 ruptured) in 671 patients using WEB in multiple centers, with a complication rate of 15.1% and a mortality rate of 3.4%. The immediate complete occlusion rate (RR1) was 43.3%, which increased to 85% at 11 months after the procedure (Adeeb et al., 2022). In addition, because only a tiny portion of the WEB device enters the parent artery, it is believed that periprocedural antiplatelet therapy is not necessary to prevent ischemic complications. Therefore, WEB is considered to be effective in treating ruptured aneurysms as well. However, a low immediate occlusion rate may have a possibility to increase the likelihood of recurrent bleeding in aneurysms that are still in the acute phase after treatment (Monteiro et al., 2022). Monteiro et al. conducted a meta-analysis of 377 ruptured aneurysms data from 9 studies using WEB, with an intraprocedural complication rate of 8.4% (95% CI 3.6 to 13.3%) and a postprocedural complication rate of 1% (95% CI 0 to 2%), respectively. The rate of adequate occlusion (RR1 + RR2) at the last follow-up was 84.8% (95% CI 73 to 96.6%; Monteiro et al., 2022).

The number of applications for other specialized devices are relatively limited, and when compared to these limited results, the performance of Comaneci/Cascade devices is also similar. Krupa et al. conducted a meta-analysis of 200 bifurcation IAs (48 ruptures) in 198 patients treated with pCONUS devices. The complication rate was 17.3% (95% CI, 10.0 to 26.2%), and the immediate RR1 rate was 46.8% (95% CI, 33.3 to 59.0%), RR2 rate was 32.9% (95% CI, 20.8 to 45.0%). The RR1 rate was 55.0% (95% CI, 43.7 to 65.6%), and the RR2 rate was 29.0% (95% CI, 19.4 to 39.4%) at 3–6 months after the procedure (Krupa et al., 2021). Pranata et al. conducted a meta-analysis of 157 patients’ data from six studies using Pulse Rider devices for the treatment of WNBAs. The incidence of complications was 5% (95% CI, 1 to 8%), the immediate RR1 + RR2 rate was 90% (95% CI, 85 to 94%), and the RR1 + RR2 rate was 91% (95% CI, 85 to 96%) at 6-month follow-up (Pranata et al., 2021). Ghozy et al. conducted a meta-analysis of 131 patients’ data from six studies using Contour devices for the treatment of bifurcation IAs. The overall incidence of adverse events was 4.70% (95% CI, 3.24 to 6.76%), and the immediate RR1 + RR2 rate was 84.21% (95% CI, 75.45 to 90.25%). At 6-month follow-up, the RR1 + RR2 rate was 91% (95% CI, 85 to 96%).

Before the Comaneci/Cascade devices can be widely applied, we believe that there are certain areas for further improvement. In this study, we noticed some cases of technical failure. Errors in size selection can be avoided, while others cannot provide sufficient support for embolization due to limited device size. Comaneci comes in three versions. The standard model Comaneci has a length of 35 mm and an expanded width of up to 4.5 mm. This device is compatible with 0.021″ microcatheters. The intermediate version, Comaneci Petit, has a length of 24 millimeters, expanded to a width of 3.5 mm, and is compatible with 0.021″ microcatheters. The Comaneci 17 has a length of 17 mm and a width of up to 3 millimeters, making it compatible with 0.021″ microcatheters (Lim et al., 2021). Cascade is compatible with 0.021″ microcatheters and comes in two sizes: M-recommended vessel diameter of 2-4 mm; L-L-recommended blood vessel diameter is 4–6 mm. Before improving the size and adaptation range of the devices, the operator should also make cautious judgments. In addition, as a retrievable device, there may be cases where the stent is caught by the spring coil. To prevent such scenarios, the operator must possess extensive expertise and build up their experience. However, our research identified instances of thrombus formation within the device or artery when antiplatelet therapy is not administered, though the majority of these cases did not progress into an ischemic stroke. This may require us to prioritize the strategy and timing of intraprocedural heparinization, although additional research is needed to determine whether antiplatelet therapy should be given during the periprocedural period. We hope to balance the possibility of ischemic events while preventing acute bleeding. Nevertheless, the Comaneci/Cascade devices have thus far offered us significant promise and protentional.

Our research still has limitations. There is no prospective data available for the studies of interest, so most of the included studies are retrospective studies with small samples and no control group, which inevitably brings bias to the evaluation. In addition, antiplatelet strategies are not the same in various studies, and the follow-up time is not consistent, which can affect the reliability of the results of this meta-analysis. Therefore, the results of the current study must be treated with caution.

## Conclusion

The Comaneci/Cascade devices are promising endovascular devices for the treatment of wide-necked and bifurcation IAs. In this meta-analysis, we have summarized all relevant studies to date and have substantiated the utilization of Comaneci/Cascade devices in the treatment of IAs has a good occlusive effect. Nevertheless, it is evident that additional equipment or technological innovation are still needed to reduce the incidence of complications. The overall results align with other types of endovascular devices. However, further prospective and randomized trials are still needed to assess the efficacy and safety of the Comaneci/Cascade devices.

## Data availability statement

The original contributions presented in the study are included in the article/Supplementary material, further inquiries can be directed to the corresponding authors.

## Author contributions

BS: Writing – original draft, Writing – review & editing. SL: Writing – original draft, Writing – review & editing. HS: Writing – review & editing. YL: Writing – review & editing. YL: Writing – review & editing. BZ: Writing – review & editing. PW: Writing – review & editing. CW: Writing – review & editing. HS: Writing – review & editing. SX: Writing – review & editing.

## Funding

The author(s) declare financial support was received for the research, authorship, and/or publication of this article. This work was supported by the National Nature Science Foundation of China (Grant no. 82071309), the Wu Jieping Medical Foundation (Grant no. 320.6750.2021-04-61), and the Research Innovation Fund of the First Affiliated Hospital of Harbin Medical University (Grant no. 2023M04).

## Conflict of interest

The authors declare that the research was conducted in the absence of any commercial or financial relationships that could be construed as a potential conflict of interest.

## Publisher’s note

All claims expressed in this article are solely those of the authors and do not necessarily represent those of their affiliated organizations, or those of the publisher, the editors and the reviewers. Any product that may be evaluated in this article, or claim that may be made by its manufacturer, is not guaranteed or endorsed by the publisher.

## Supplementary material

SUPPLEMENTARY FIGURE S1 Estimated plotted rates of immediate complete occlusion (defined as Raymond-Roy class 1).

SUPPLEMENTARY FIGURE S2 Estimated plotted rates of complete occlusion at last follow-up.

SUPPLEMENTARY FIGURE S3 Funnel plot for evaluating technical success publication bias.

SUPPLEMENTARY FIGURE S4 Funnel plot for evaluating publication bias in periprocedural applications.

SUPPLEMENTARY FIGURE S5 Funnel plot for evaluating the publication bias of immediate adequate occlusion.

SUPPLEMENTARY FIGURE S6 Funnel plot for evaluating the publication bias of adequate occlusion at the last follow-up.

SUPPLEMENTARY FIGURE S7 Funnel plot for evaluating the publication bias of immediate complete occlusion.

SUPPLEMENTARY FIGURE S8 Funnel plot for evaluating the publication bias of complete occlusion at the last follow-up.
