# Supplementary figures and images for: A systematic review and meta-analysis of Comaneci/Cascade temporary neck bridging devices for the treatment of intracranial aneurysms
Source: Front Hum Neurosci. 2023 Sep 25;17:1276681. doi: 10.3389/fnhum.2023.1276681 (PMC10560715; doi:10.3389/fnhum.2023.1276681)

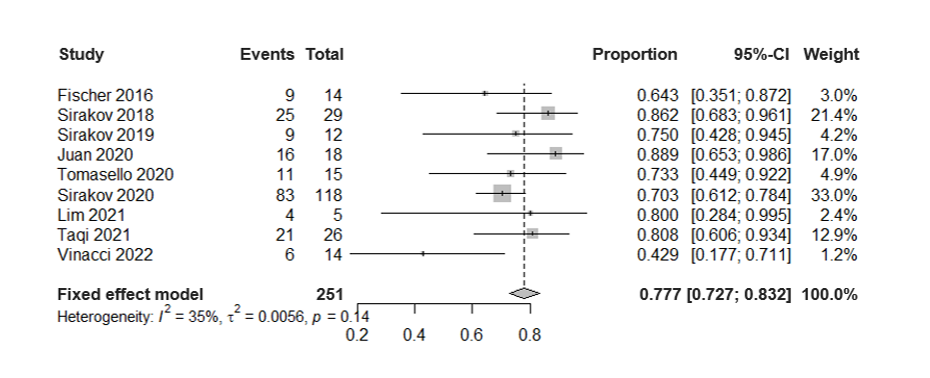

Supplement: SUPPLEMENTARY FIGURE S1 — Estimated plotted rates of immediate complete occlusion (defined as Raymond-Roy class 1). [file Image_1.TIF]

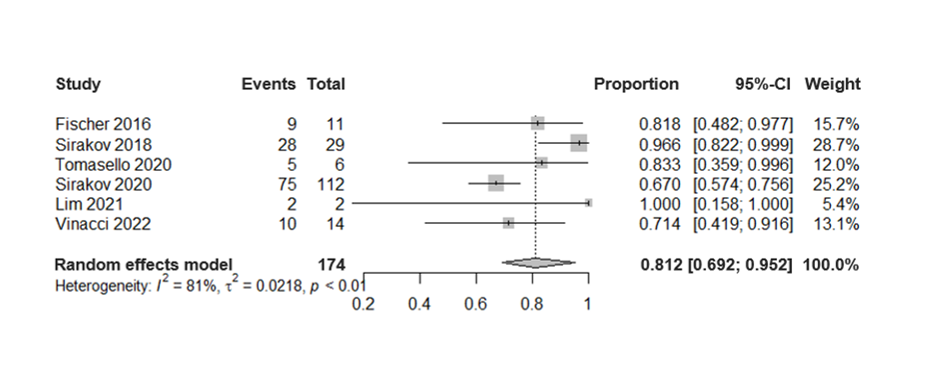

Supplement: SUPPLEMENTARY FIGURE S2 — Estimated plotted rates of complete occlusion at last follow-up. [file Image_2.TIF]

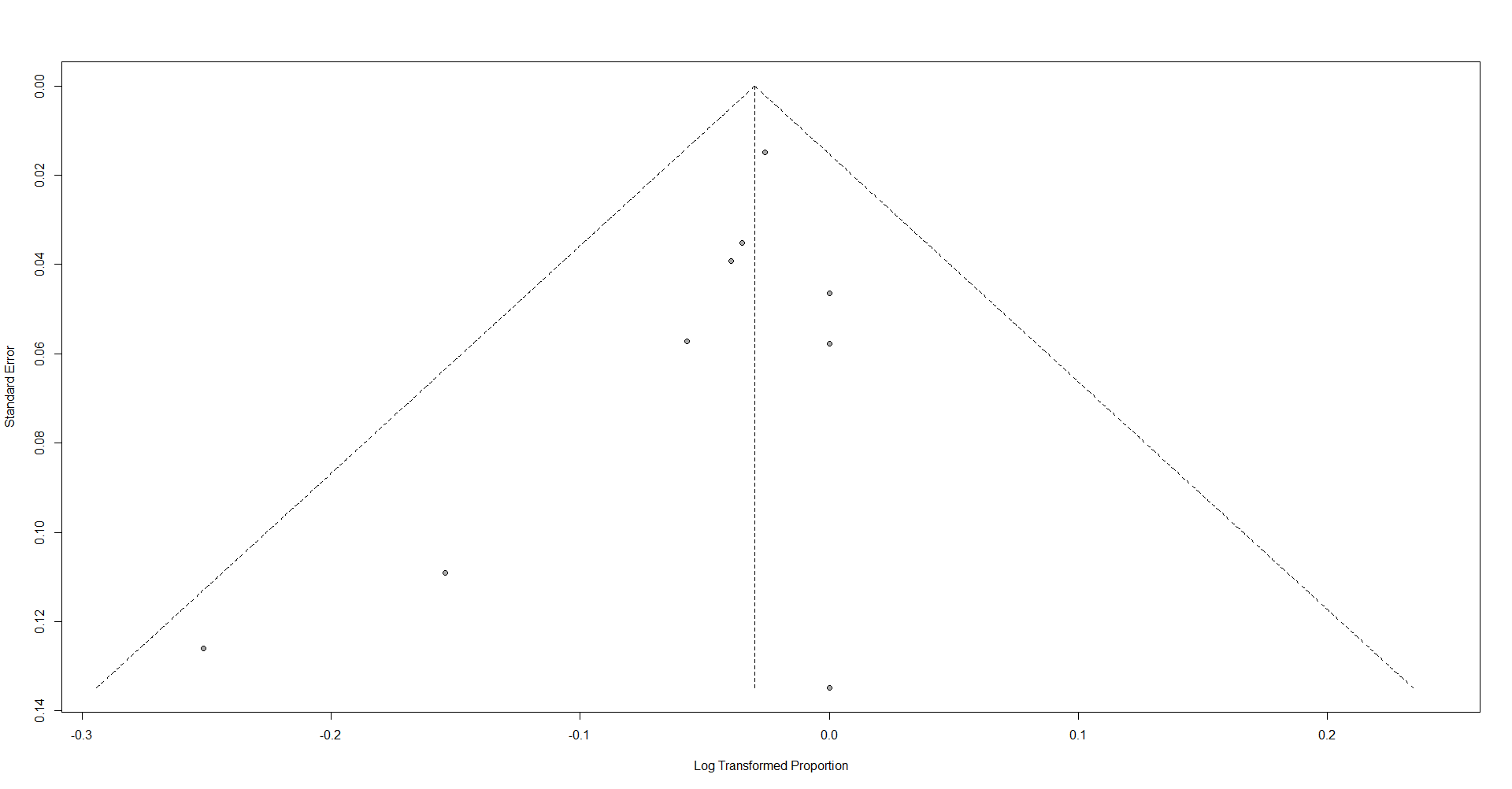

Supplement: SUPPLEMENTARY FIGURE S3 — Funnel plot for evaluating technical success publication bias. [file Image_3.TIFF]

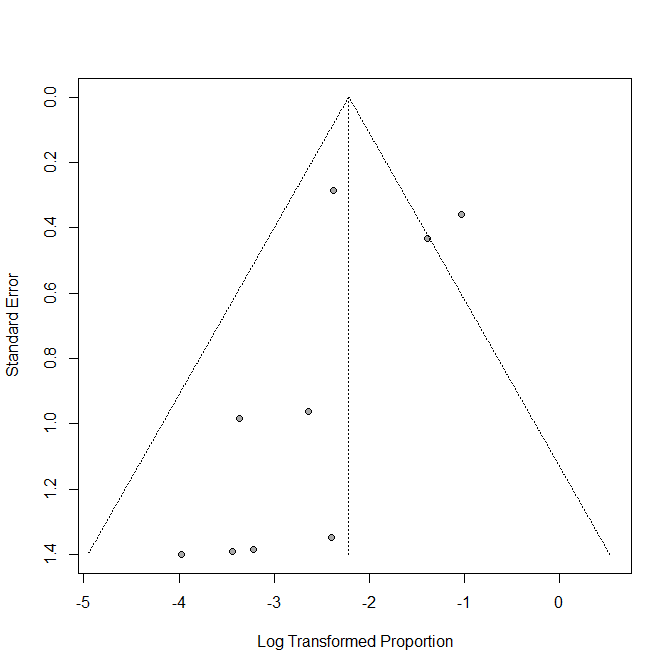

Supplement: SUPPLEMENTARY FIGURE S4 — Funnel plot for evaluating publication bias in periprocedural applications. [file Image_4.TIFF]

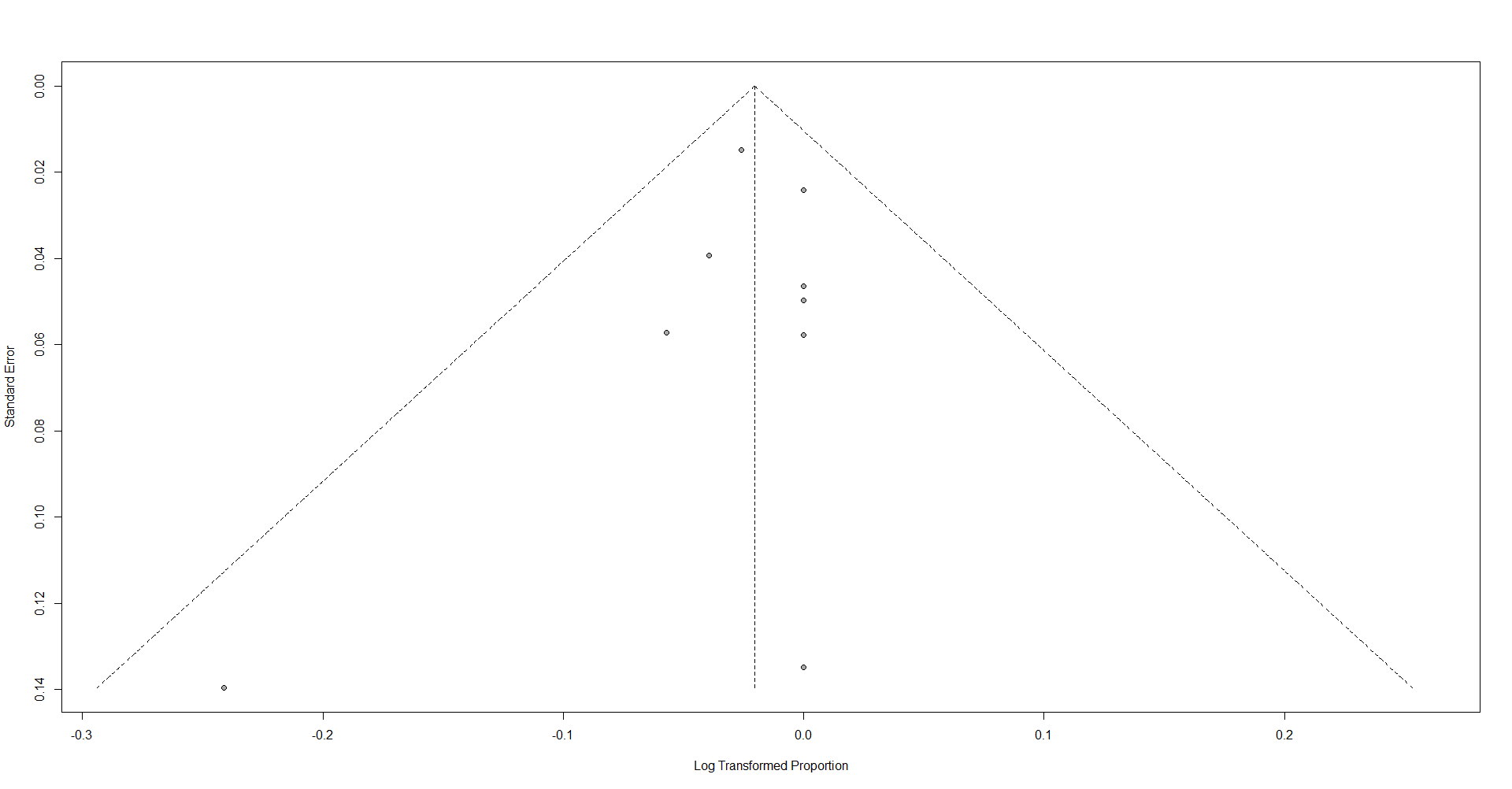

Supplement: SUPPLEMENTARY FIGURE S5 — Funnel plot for evaluating the publication bias of immediate adequate occlusion. [file Image_5.TIFF]

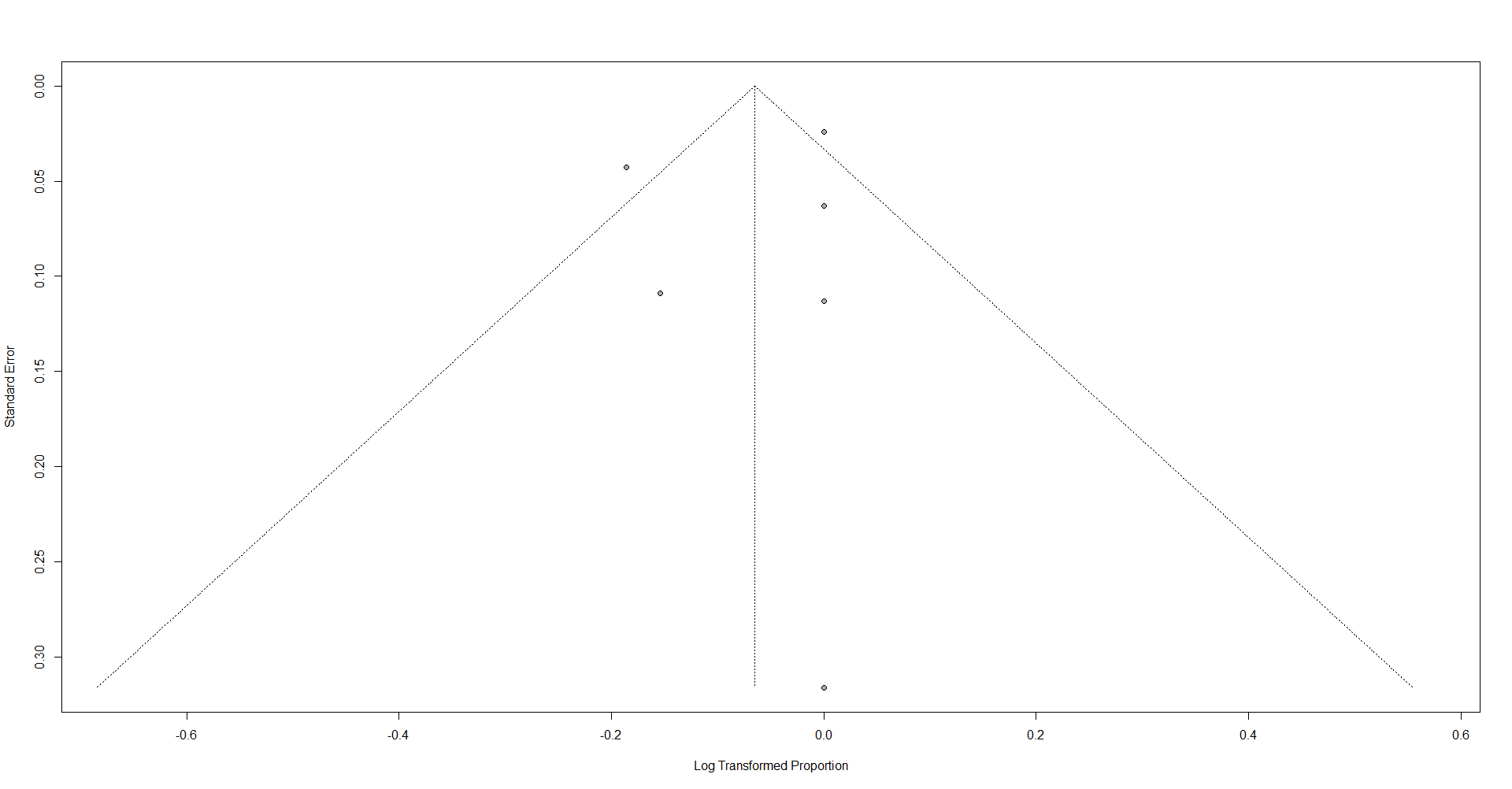

Supplement: SUPPLEMENTARY FIGURE S6 — Funnel plot for evaluating the publication bias of adequate occlusion at the last follow-up. [file Image_6.TIFF]

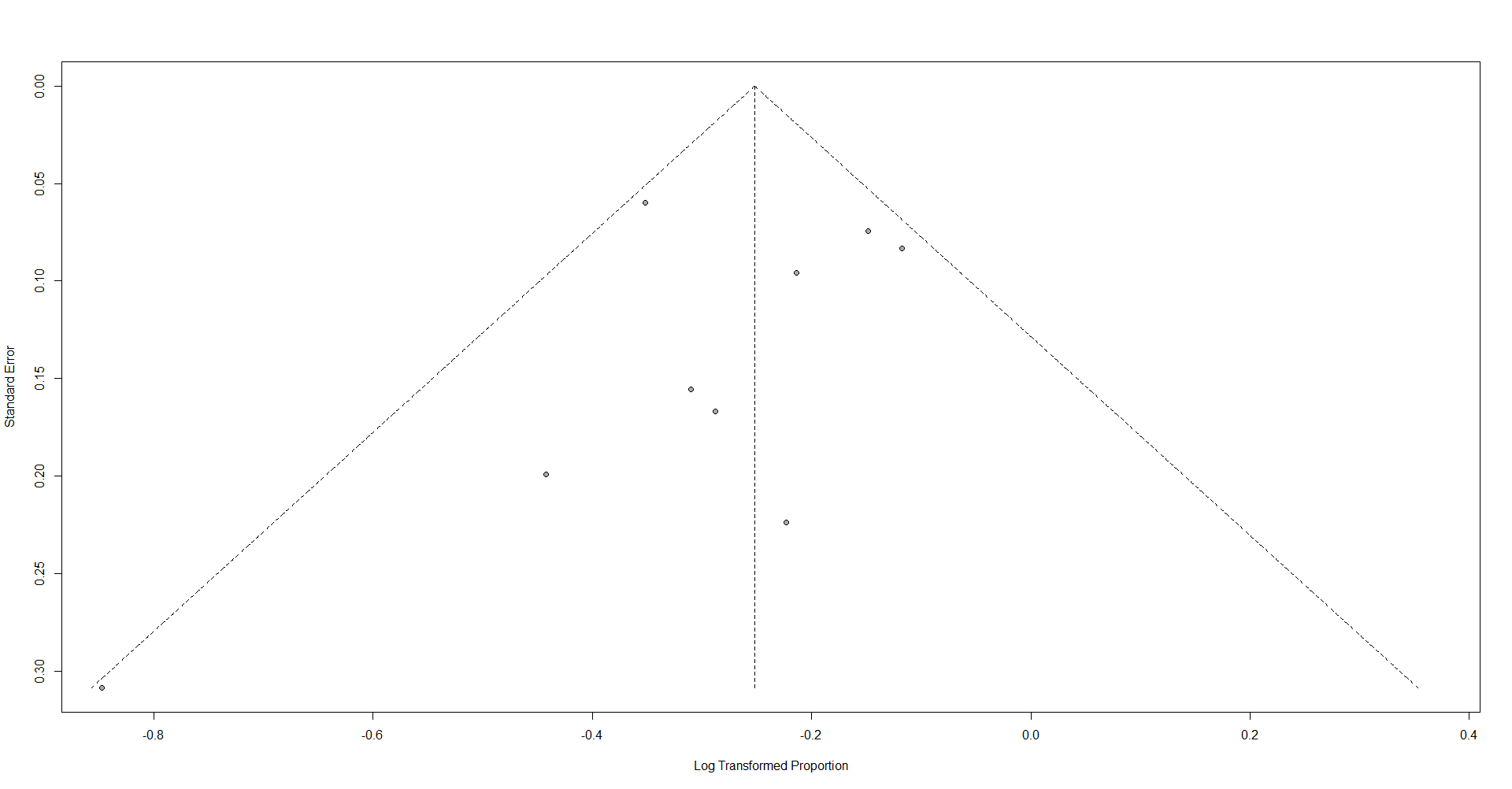

Supplement: SUPPLEMENTARY FIGURE S7 — Funnel plot for evaluating the publication bias of immediate complete occlusion. [file Image_7.TIFF]

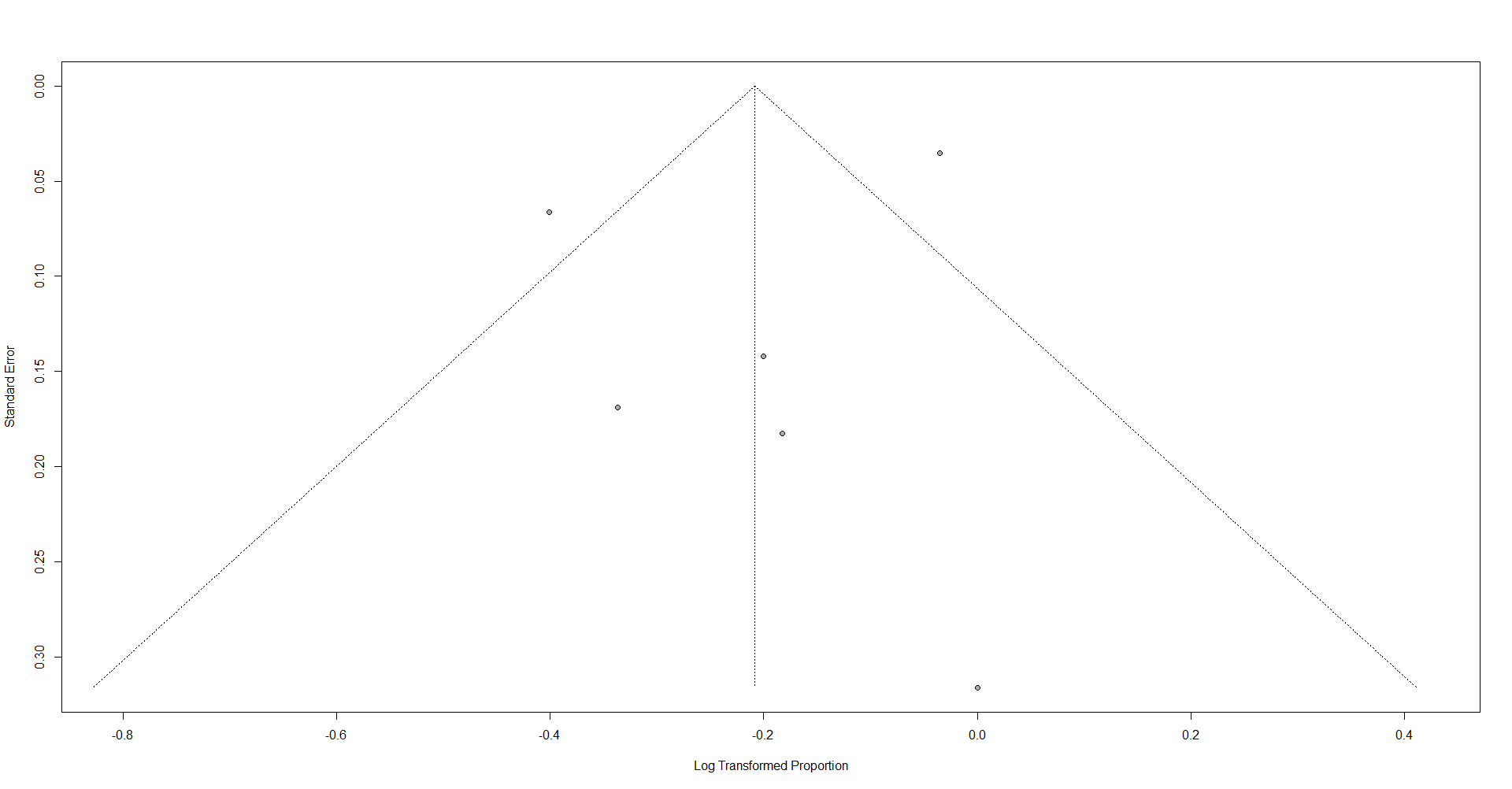

Supplement: SUPPLEMENTARY FIGURE S8 — Funnel plot for evaluating the publication bias of complete occlusion at the last follow-up. [file Image_8.TIFF]
