# Supplementary material for: A systematic review and meta-analysis of Comaneci/Cascade temporary neck bridging devices for the treatment of intracranial aneurysms
Source: Front Hum Neurosci. 2023 Sep 25;17:1276681. doi: 10.3389/fnhum.2023.1276681 (PMC10560715; doi:10.3389/fnhum.2023.1276681)
Supplement: Supplementary file 9 [file Table_1.docx]

**Table S1. Search syntax**

| PubMed Search Accessed on April 21, 2023  (62 Articles) | EMBASE Search Accessed on April 21, 2023 (146 Articles) | Web of Science Search Accessed on April 21, 2023 (83 Articles) |
| --- | --- | --- |
| #1 intracranial aneurysm [Mesh]  #2 intracranial aneurysm [Title/Abstract]  #3 intracranial aneurysms [Title/Abstract]  #4 intracerebral aneurysm [Title/Abstract]  #5 intracerebral aneurysms [Title/Abstract]  #6 cerebral aneurysm [Title/Abstract]  #7 cerebral aneurysms [Title/Abstract]  #8 brain aneurysm [Title/Abstract]  #9 brain aneurysms [Title/Abstract]  #10 #1 OR #2 OR #3 OR #4 OR #5 OR #6 OR #7 OR #8 OR #9  #11 temporary neck bridging [Title/Abstract]  #12 Comaneci [Title/Abstract]  #13 Cascade [Title/Abstract]  #14 #11 OR #12 OR #13  #15 #10 AND #14 | #1 ‘intracranial aneurysm’: ab,ti  #2 ‘intracranial aneurysms’: ab,ti  #3 ‘intracerebral aneurysm’: ab,ti  #4 ‘intracerebral aneurysms’: ab,ti  #5 ‘cerebral aneurysm’: ab,ti  #6 ‘cerebral aneurysms’: ab,ti  #7 ‘brain aneurysm’: ab,ti  #8 ‘brain aneurysms’: ab,ti  #9 #1 OR #2 OR #3 OR #4 OR #5 OR #6 OR #7 OR #8  #10 ‘temporary neck bridging’: ab,ti  #11 ‘Comaneci’: ab,ti  #12 ‘Cascade’: ab,ti  #13 #10 OR #11 OR #12  #14 #9 AND #13 | #1 TS= (intracranial aneurysm OR intracranial aneurysms OR intracerebral aneurysm OR intracerebral aneurysms OR cerebral aneurysm OR cerebral aneurysms OR brain aneurysm OR brain aneurysms)  #2 TS= (temporary neck bridging OR Comaneci OR Cascade)  #3 #1 AND #2 |
