# Supplementary material for: A systematic review and meta-analysis of Comaneci/Cascade temporary neck bridging devices for the treatment of intracranial aneurysms
Source: Front Hum Neurosci. 2023 Sep 25;17:1276681. doi: 10.3389/fnhum.2023.1276681 (PMC10560715; doi:10.3389/fnhum.2023.1276681)
Supplement: Supplementary file 10 [file Table_2.docx]

**Supplementary Table 2. The quality measure of included studies by the Modified Newcastle-Ottawa Quality Assessment Scale: retrospective design**

|  | **Selection** | | | |  | **Outcome** | |  |
| --- | --- | --- | --- | --- | --- | --- | --- | --- |
| **Study Name** | **1** | **2** | **3** | **4** | **Comparability** | **1** | **2** | **Total** |
| Fischer 2016 | * | * | c | b | c | * | * | 4 |
| Sirakov 2018 | * | * | c | * | c | * | * | 5 |
| Sirakov 2019 | * | * | c | * | c | * | b | 4 |
| Juan 2020 | * | * | c | * | c | * | * | 5 |
| Tomasello 2020 | * | * | c | * | c | * | * | 5 |
| Sirakov 2020 | * | * | c | * | c | * | * | 5 |
| Lim 2021 | * | * | c | * | c | * | * | 5 |
| Taqi 2021 | * | * | c | b | c | * | b | 3 |
| Vinacci 2022 | * | * | c | * | c | * | * | 5 |

**Selection**

1) Is the case definition adequate?

a) Yes, with independent validation*

b) Yes, eg, record linkage or based on self-reports

c) No description

2) Representativeness of the cases

a) Consecutive or obviously representative series of cases*

b) Potential for selection biases or not stated

3) Selection of controls

a) Community controls*

b) Hospital controls

c) No description

4) Definition of controls

a) No history of disease (end point) *

b) No description of source

**Comparability**

1) Comparability of cases and controls based on the design or analysis

a) Study controls for _____ (select the most important factor) *

b) Study controls for any additional factor (these criteria could be modified to indicate a specific control for a second important factor) *

b) No comparability

**Exposure**

1) Assessments of exposure

a) Secure record (eg, surgical records) *

b) Structured interview where blind to case/control status*

c) Interview not blinded to case/control status

d) Written self-report or medical record only

e) No description

2) Sufficient follow-up time

a) Yes*

b) No

Note: A study can be awarded a maximum of one * for each numbered item within the Selection and Outcome categories. A maximum of two * can be given for Comparability. Studies with ≥6 * positive answers were defined as good quality.
