# Supplementary material for: A systematic review and meta-analysis of Comaneci/Cascade temporary neck bridging devices for the treatment of intracranial aneurysms
Source: Front Hum Neurosci. 2023 Sep 25;17:1276681. doi: 10.3389/fnhum.2023.1276681 (PMC10560715; doi:10.3389/fnhum.2023.1276681)
Supplement: Supplementary file 11 [file Table_3.docx]

**Supplementary Table 3.** Details of technical failure.

| **Author, year** | **Technical Failure** |
| --- | --- |
| Fischer *et al,*  2016 | 4/18 (22.2%)  In 4 cases a stable position of the Comaneci device with proper expansion and coverage of the vessel wall and the aneurysm orifice could not be achieved. The treatment strategy was changed to stent-assisted embolization in 2 cases, and the remaining 2 cases were treated using the remodeling technique, with a compliant balloon. |
| Sirakov *et al,*  2018 | 1/29 (3.45%)  The failure occurred due to the severe vasospasm; the device was not fully opened but provided enough coverage of the neck to provide protection of the parent artery. |
| Sirakov *et al,*  2019 | 0/12 (0%)  No technical failure. |
| Juan *et al,*  2020 | 1/18 (6.2%)  The failure occurred due to a wrong choice of the device, as it was a ruptured fusiform carotid aneurysm. Adequate apposition of the device to the ill vessel or coil disposition stability were not achieve, so the treatment was dismissed and the case was retreated using a flow diverter stent. |
| Tomasello *et al,* 2020 | 0/15 (0%)  No technical failure. |
| Sirakov *et al,*  2020 | 3/118 (2.54%)  All failure occurred due to insufficient neck coverage caused by undersizing of the Comaneci device selected by the main operator due to planning and measurement errors. |
| Lim *et al,*  2021 | 0/5 (0%)  No technical failure. |
| Taqi *et al,*  2021 | 1/26 (3.8%)  Two Comaneci 17 devices were placed in the superior and inferior division of the middle cerebral artery. Upon retrieval of the 2 devices, one of the coil loops was attached to the device and continued to pull through the aneurysm. The removal resulted in the movement of the positioned coils out of the aneurysm and into the parent vessel. This event was treated with the placement of 2 Atlas stents with excellent result and no adverse outcome. |
| Vinacci *et al,*  2022 | 2/14 (14%)  The failure occurred due to Comaneci device did not achieve a satisfying scaffolding effect on the neck of the aneurysm and a self-expandable stent was required. |
