# Supplementary material for: A systematic review and meta-analysis of Comaneci/Cascade temporary neck bridging devices for the treatment of intracranial aneurysms
Source: Front Hum Neurosci. 2023 Sep 25;17:1276681. doi: 10.3389/fnhum.2023.1276681 (PMC10560715; doi:10.3389/fnhum.2023.1276681)
Supplement: Supplementary file 12 [file Table_4.docx]

**Supplementary Table 4.** Details of adverse events.

| **Author, year** | **Adverse events** |
| --- | --- |
| Fischer *et al,*  2016 | 1 case (7.14%).  In the case the Comaneci could not be completely ‘deflated’ before removal, and it was therefore withdrawn without protection of the microcatheter. The patient developed a left-sided hemiparesis within 3 hours after procedure. DSA disclosed a thrombotic occlusion of the target ICA that was successfully recanalized by MT. The patient’s clinical condition improved significantly to a mRS grade of 1, with a remaining mild weakness of the left arm at discharge. |
| Sirakov *et al,*  2018 | 1 case (3.45%).  While withdrawing the device into the microcatheter, there were some noticeable changes in the coil structure inside the aneurysmal. The patient developed cerebellar ataxia 24 hours after the procedure. Analysis of this case lead to the conclusion that a stable basket of coils was not achieved, and that this led to coil protrusion, compromising the origin of the left superior cerebellar artery (SCA). |
| Sirakov *et al,*  2019 | No complications. |
| Juan *et al,*  2020 | 4 cases (25%)  3 minor complications: appearance of platelet aggregates in two cases, which was solved with antiaggregant agents during the procedure, and one case of coil migration which is solved satisfactorily using the Comaneci device as extractor, and the coil could be withdrawn without causing any ischemic or hemorrhagic lesion.  1 major complication: ischemic stroke in the territory of the AChoA after embolization of a giant aneurysm in the PComA. Inadvertent partial occlusion of the origin of the AChoA by Comaneci filaments during deployment, distortion of the flow caused by the device or compressive events on the AChoA derived from embolization itself should probably be considered the cause of ischemia. This patient was treated with aspirin 100 mg once the infarction was demonstrated, and his motor deficit progressed favorably and with very few sequels. |
| Tomasello *et al,*  2020 | No complications |
| Sirakov *et al,*  2020 | 11 cases (8.73%).  7 cases: periprocedural thromboembolic complications related to the device. In these cases, small thrombus formation across the distal part of the device and emboli in the distal vascular distribution of the parent vessel were noted.  After injection of a total of 10 mg Reopro abciximab, the thrombi dissolved in all cases. These events lead to permanent neurological deficit in 3 patients.  5 cases: Vascular sequelae in terms of severe vasospasm of the parent artery occurred after complete withdrawal of the Comaneci caused by possible overexpansion of the mesh and flanking of the distal tip of the device.  1 case overlapping. |
| Lim *et al,*  2021 | No complications |
| Taqi *et al,*  2021 | No complications |
| Vinacci *et al,*  2022 | 5 cases (35.7%)  All cases had clot formation inside the mesh of the Comaneci device, 1 of them had parent artery clot formation. All patients received antiplatelet therapy (aspirin and tirofiban) or stent rescue measures, and no permanent complications were found. |

DSA: digital subtraction angiography; ICA: Internal carotid artery; MT: mechanical thrombectomy; mRS: modified Rankin scale; AChoA: anterior choroidal artery; PcomA: posterior communicating artery.
