# Supplementary material for: A systematic review and meta-analysis of Comaneci/Cascade temporary neck bridging devices for the treatment of intracranial aneurysms
Source: Front Hum Neurosci. 2023 Sep 25;17:1276681. doi: 10.3389/fnhum.2023.1276681 (PMC10560715; doi:10.3389/fnhum.2023.1276681)
Supplement: Supplementary file 13 [file Table_5.docx]

**Supplementary Table 5.** Details of reoccurrence and retreatment

| **Author, year** | **Follow-up** **Reoccurrence and Retreatment** |
| --- | --- |
| Fischer *et al,*  2016 | None. |
| Sirakov *et al,*  2018 | None. |
| Sirakov *et al,*  2019 | NA |
| Juan *et al,*  2020 | 2 years:1 case of aneurysm regrowth. The case presented a new episode of SAH 2 years after the first embolization and was retreated using a flow diverter. |
| Tomasello *et al,*  2020 | 6 months: 1 patient developed a residual neck at follow-up despite having immediate complete occlusion. |
| Sirakov *et al,*  2020 | Follow-up at a mean of 81.1d: 13 (11.6%) showed signs of aneurysmal recanalization in terms of a decrease in the RR classification.  Follow-up at 6-mo at a mean of 165.1d: Progressive aneurysmal recanalization was noted in 19/112 patients (16.94%).  Total recanalization rate of 14.28%. Following embolization via either a flow redirecting device or an additional coil embolization was scheduled for all 37 patients |
| Lim *et al,*  2021 | 1 case with the ruptured posterior inferior cerebellar artery aneurysm: The patient’s neurological examination did not improve, and the patient’s family opted for comfort care measures and withdrawal of care. |
| Taqi *et al,*  2021 | NA |
| Vinacci *et al,*  2022 | In a range of 12–18 months, 1 case had revascularization of the aneurysm sack and required the deployment of a flow diverter stent. |

NA, not available; SAH, subarachnoid hemorrhage; RR: Ray-Raymond.
